# The Effect of Propofol versus Sevoflurane on Postoperative Delirium in Parkinson’s Disease Patients Undergoing Deep Brain Stimulation Surgery: An Observational Study

**DOI:** 10.3390/brainsci12060689

**Published:** 2022-05-25

**Authors:** Yongde Zhou, Zhengqian Li, Yu Ma, Cuiping Yu, Yao Chen, Jian Ding, Jianfeng Yu, Rongsong Zhou, Xiaoxiao Wang, Taotao Liu, Xiangyang Guo, Ting Fan, Chengmei Shi

**Affiliations:** 1 Department of Anesthesiology, Tsinghua University Yuquan Hospital, Beijing 100040, China; 15810362998@163.com (Y.Z.); ziboycp@sohu.com (C.Y.); chenyao628@163.com (Y.C.); dj32816@126.com (J.D.); 15210079723@163.com (J.Y.); 2 Department of Anesthesiology, Peking University Third Hospital, Beijing 100191, China; zhengqianli@bjmu.edu.cn (Z.L.); liutaotao1101@163.com (T.L.); puthmzk@hsc.pku.edu.cn (X.G.); 3 Beijing Center of Quality Control and Improvement on Clinical Anesthesia, Beijing,100191, China; 4 Department of Neurosurgery, Tsinghua University Yuquan Hospital, Beijing 100040, China; mayu@tsinghua.edu.cn (Y.M.); rongsong0925@163.com (R.Z.); 5 Research Center of Clinical Epidemiology, Peking University Third Hospital, Beijing 100191, China; wxx910129@163.com

**Keywords:** postoperative delirium, Parkinson’s disease, anesthesia maintenance, propofol, sevoflurane

## Abstract

Background: The selection of the maintenance of general anesthesia may affect the development of postoperative delirium (POD), notably for Parkinson’s disease (PD) patients, due to their lower cognitive reserve. The present study was designed to compare the potential impact of propofol vs. sevoflurane based general anesthesia maintenance methods on the development of POD in PD patients following deep brain stimulation (DBS) surgery. Methods: A total of 125 PD patients who were scheduled to undergo DBS surgery were randomly divided into the propofol (*n* = 63) and the sevoflurane groups (*n* = 62). The patients in the two groups randomly received propofol- or sevoflurane-based general anesthesia. The Confusion Assessment Method (CAM) was employed by an investigator who was blinded to the anesthesia regimen and was administered twice per day from postoperative day 1 until discharge. Results: The incidence of POD was 22.22% (14/63) with propofol anesthesia and 20.97% (13/62) with sevoflurane anesthesia (*p =* 0.865). In addition, no difference was noted in the duration and severity of delirium between the propofol and sevoflurane groups. Conclusions: In the present study, propofol- and sevoflurane-based general anesthesia exhibited comparable results with regard to the POD incidence in PD patients undergoing deep brain stimulation surgery.

## 1. Introduction

Parkinson’s disease (PD) is a common neurodegenerative disease. Its main clinical symptoms include static tremors, muscle rigidity, and bradykinesia [1]. Deep brain stimulation (DBS) of the subthalamic nuclei is a standard treatment option for advanced PD patients. Bilateral STN-DBS improves motor and a variety of non-motor symptoms [1,2], as well as health-related quality of life [3,4]. DBS can also reduce the levodopa medication dose and ameliorate the side effects associated with levodopa therapy [5].

Nevertheless, postoperative delirium (POD) is a common complication following deep brain stimulation surgery for PD patients, with an incidence range of 19.4% to 42.6% [2,5]. POD is an acute disorder of attention and cognition in elderly people that is common, serious, costly, under-recognized, and often fatal [6]. POD has been independently associated with worsened clinical outcomes, increased costs, and increased mortality in patients [6]. 

PD is a common neurodegenerative disease and POD is common in patients who are treated with DBS surgery [2]. A limited number of studies have assessed the occurrence of POD in PD patients following DBS surgery under general anesthesia, and the incidence range reported was 19.4–42.6% [2,5]. Unfortunately, these studies did not provide a detailed description of the general anesthesia regimens. At present, it is not clear which type of general anesthesia is beneficial to reduce the incidence of POD. With regard to the PD patients, the influence of the anesthesia maintenance method on the incidence, severity, and duration of POD is currently unclear. In the present study, the risk of POD was examined in PD patients who were under propofol and sevoflurane maintenance conditions.

Propofol-based total intravenous anesthesia and sevoflurane-based inhalational anesthesia are the common anesthesia maintenance methods used in clinical practice. The use of propofol-based anesthesia has been associated with reduced postoperative nausea and vomiting and atmospheric pollution as well as a reduced chance of triggering malignant hyperthermia [7]. Sevoflurane-based inhalational anesthesia is known for bronchodilation, pre- and post-ischemic conditioning, lower costs, and ease of delivery and use [1]. Previous evidence suggests that the choice of anesthetics may also influence postoperative cognitive status. Certain studies have shown that compared with propofol, sevoflurane damages cognitive status. For example, Ishii et al. reported in their study that in comparison with sevoflurane anesthesia, propofol anesthesia was associated with a lower incidence of POD in elderly patients [8]. Tang et al. reported that the negative cognitive effects were more severe following sevoflurane anesthesia than those noted following propofol anesthesia [9]. Conversely, several studies have shown the opposite conclusions. Nishikawa et al. indicated that the score of the delirium rating scale was lower in the sevoflurane group than that noted in the propofol group on postoperative days 2 and 3 [10]. Schoen et al. demonstrated that postoperative cognitive function was improved in the sevoflurane group compared with that of the propofol group [11]. Zhang et al. indicated that the use of propofol for general anesthesia was associated with a decreased delayed neurocognitive recovery in older adults compared with the use of sevoflurane [12].

This prospective observational study was conducted to identify the incidence, severity, and duration of POD in PD patients undergoing DBS surgery with different anesthesia maintenance methods.

## 2. Materials and Methods

### 2.1. Study Design

This was a prospective, randomized study. The research proposal was approved by the Ethics Committee of Yuquan Hospital of Tsinghua University (No. 20190014). All patients enrolled signed the relevant informed consent forms. The clinical trial registration was completed prior to patient enrollment (ChiCTR1900027210).

### 2.2. Sample Size Estimation

Based on previous studies [5,13], a sample of 110 patients was selected (55 in group 1; 55 in group 2), which would provide the study with 80% power to detect a significant difference between the group proportions of −24% at a two-sided alpha value of 0.05. The proportion in group 1 (the propofol group) was assumed to be 19% and the proportion in group 2 (the sevoflurane group) was 43%. Given an anticipated dropout rate of 10%, the total sample size required was 124 (62 in group 1; 62 in group 2).

### 2.3. Patient Collection

**Figure 1:**
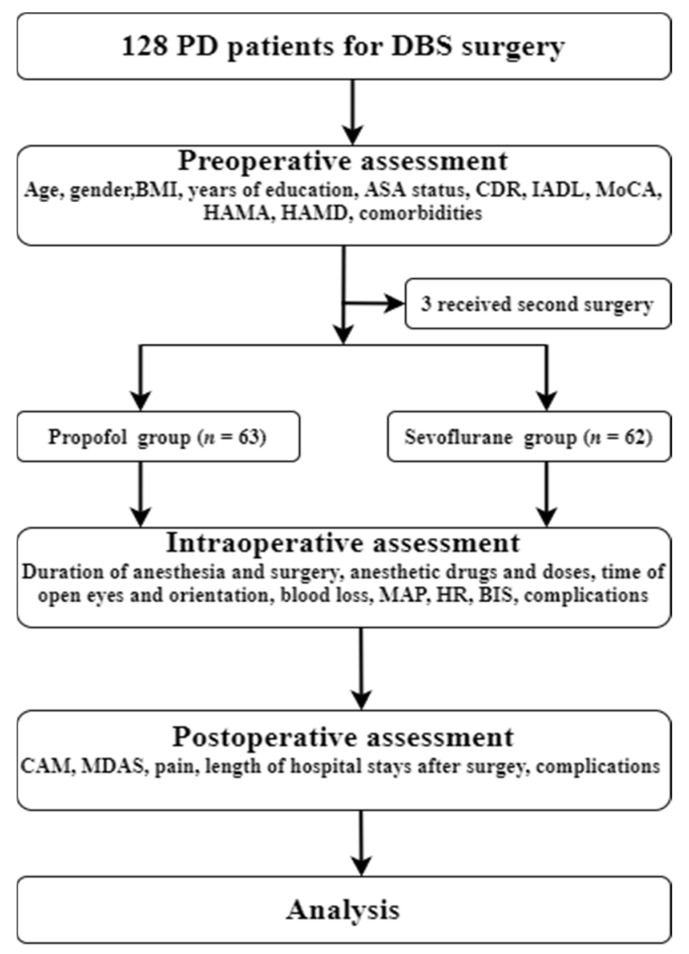
Flow chart of the trial. PD, Parkinson’s disease; DBS, deep brain stimulation; BMI, Body Mass Index; ASA, American Society of Anesthesiologists; CDR, Clinical Dementia Rating; IADL, Instrumental Activity of Daily Living; MoCA, Montreal Cognitive Assessment, HAMA, Hamilton anxiety; HAMD, Hamilton depression; MAP, mean arterial pressure; HR, heart rate; BIS, Bispectral Index; CAM, CAM, Confusion Assessment Method; MDAS, Memorial Delirium Assessment Scale.

The present study was conducted at Yuquan Hospital of Tsinghua University (Beijing, China) between November 2019 and March 2021. Eligible patients were diagnosed with PD according to the UK Brain Bank criteria [14] and treated with bilateral STN-DBS. Bilateral STN-DBS treatment was initiated according to the Movement Disorders Society guidelines [15]. According to *Chinese deep brain stimulation therapy for Parkinson’s disease Expert Consensus (Second Edition)* [16], the inclusion criteria for performing DBS surgery are: (1) primary PD, hereditary PD, or various genotypes PD, responds well to compound levodopa; (2) drug efficacy has decreased significantly, or obvious motor complications affect the patient’s quality of life; (3) adverse drug reactions that cannot be tolerated and affect the efficacy of drugs; (4) tremors that cannot be controlled by drugs. Contraindications for performing DBS surgery are: (1) significant cognitive impairment; (2) severe (refractory) depression, anxiety, schizophrenia, and other mental diseases; (3) medical comorbidities that affect surgery or survival. After reviewing patient medical records, patients were excluded if they: (1) were unable to read or had severe visual or auditory deficits; (2) had a history of alcohol abuse and drug dependence; or (3) were unwilling to comply with the study protocol or procedures [17]. A total of 128 adults were invited to participate in this study (Figure 1, flow diagram).

Preoperatively, the neuropsychiatric and neuropsychological assessments of the patients were performed, including Clinical Dementia Rating (CDR) scores, Instrumental Activity of Daily Living (IADL) scores, Montreal Cognitive Assessment (MoCA) scores, Hamilton anxiety (HAMA) scores, and Hamilton depression (HAMD) scores.

The baseline information, such as age, gender, Body Mass Index (BMI), years of education, American Society of Anesthesiologists (ASA) status, and preoperative comorbidities, was recorded.

### 2.4. Anesthesia Method

The general anesthesia and surgery were operated by a specific team to avoid interfering factors. Following transfer of the patients to the operating room, a series of measurements were performed including electrocardiograph, non-invasive blood pressure, heart rate (HR), saturation of pulse oximetry, and Bispectral Index (BIS). The induction drugs used were as follows: Sufentanil (0.3 µg/kg), propofol (1.0–2.0 mg/kg), etomidate (0.2–0.3 mg/kg), and cisatracurium (0.2 mg/kg). Following induction, a 7.5# (female) or 8.0# (male) endotracheal tube was intubated.

During the anesthesia maintenance stage, the patients randomly received total intravenous anesthesia (propofol group) or combined intravenous and inhalation anesthesia (sevoflurane group). The anesthetics used for the propofol group were propofol (4.0–8.0 mg/kg^–1^/h^−1^) and remifentanil (0.1–0.4 µg/kg^–1^ h^−1^), whereas sevoflurane (1–1.5%) and remifentanil (0.1–0.4 µg/kg^–1^ h^−1^) were used in the sevoflurane group. All patients received BIS (BIS 40–60) monitoring to adjust the anesthesia depth [18]. Vasoactive drugs were used to maintain hemodynamic stability if necessary. The systemic blood pressure was adjusted to be higher than 90 mm Hg. Following surgery, all patients in the two groups received the same analgesic treatment (sufentanil 2 µg/kg + dexmedetomidine 2.3 µg/kg diluted to 100 mL). The background infusion rate was 2 mL/h, the dosage of PCA was 0.5 mL, and the locking time was 15 min. 

The following information was recorded: time of anesthesia, operation, open eyes and orientation, the dosage of remifentanil and cisatracurium, intraoperative fluid volume, hypotension, bradycardia, postoperative nausea and vomiting (PONV), and other side effects. In addition, the Visual Analog Scale (VAS) scores following surgery and the length of stay in the hospital following surgery were recorded. The intensity of postoperative pain was evaluated twice daily at 7:00 am and 7:00 pm with the VAS. The VAS pain scale ranged from 0–10 with 0 corresponding to “no pain” and 10 to the “worst possible pain”, the number patients pointed out to indicate the pain intensity [19].

### 2.5. Operation

All patients included in the study were diagnosed with PD and met DBS indications.

The patients underwent two steps of surgery in the present study. In the first step, the patients underwent stereotactic implantation of DBS electrodes in the subthalamic nucleus (STN). The anesthesia method usually was local anesthesia with minimal sedation. Subsequently, the patients underwent imaging examination to confirm the place of the electrodes.

The second step was conducted following the imaging confirmation and the DBS batteries and leads were placed. The second step was performed under general anesthesia. The DBS generator was implanted in the sub-clavicular region and the extension wires were tunneled through the neck and connected to the DBS electrode. The patients returned to the ward following extubation.

### 2.6. Delirium Assessment

The incidence of POD was assessed by CAM [6] and its severity was assessed by the Memorial Delirium Assessment Scale (MDAS) [20].

CAM and MDAS were employed by a psychiatrist investigator who was blinded to the anesthesia regimen. The assessment of POD was performed twice a day (7:00 a.m. and 7:00 p.m.) from the first postoperative day until discharge.

### 2.7. Statistical Analysis

The IBM SPSS program for Windows (version 26) was used for statistical analysis. The one-sample Kolmogorov–Smirnov method was used to assess the normality of all variables. Normally distributed variables were presented as mean ± standard deviation (SD) and analysis was performed using a Student’s *t*-test. Non-normally distributed variables were presented as median (interquartile spacing) and analysis was performed using the Mann–Whitney U test. Normally distributed variables included age, BMI, years of education, IADL scores, MoCA scores, mean BIS value, estimated blood loss, length of surgery, length of anesthesia, the dosage of remifentanil and cisatracurium, open eye time, VAS scores, orientation time, and length of stay in hospital following surgery. Non-normally distributed variables included the CDR scores, HAMA scores, HAMD scores, MADS score, and duration of POD. The categorical factors, such as gender, ASA classification, preoperative comorbidities, intraoperative hypotension, bradycardia, PONV, and POD were presented as the number and percentage of participants. The categorical variables were analyzed using the chi-square test or the Fisher’s exact test. A *p* value lower than 0.05 was considered to indicate a statistically significant difference.

## 3. Results

### 3.1. Baseline Characteristics

A total of 63 and 62 patients were included in the propofol and sevoflurane groups, respectively. No significant differences were noted between the propofol and sevoflurane groups with regard to age, gender, BMI, years of education, ASA status, preoperative comorbidities, CDR scores, IADL scores, MoCA scores, HAMA scores, and HAMD scores (Table 1).

### 3.2. Characteristics of Anesthesia and Surgery

All 125 patients received the same surgical treatment. No significant differences were noted between the propofol and sevoflurane groups with regard to the mean BIS values, estimated blood loss, length of surgery, the dosage of remifentanil and cisatracurium, and the incidence of hypotension and bradycardia during the surgery. Following surgery, no significant differences were noted between the propofol and sevoflurane groups with regard to the incidence of PONV, the VAS scores, and the length of stay in the hospital (Table 2).

### 3.3. Hemodynamic Values

**Figure 2:**
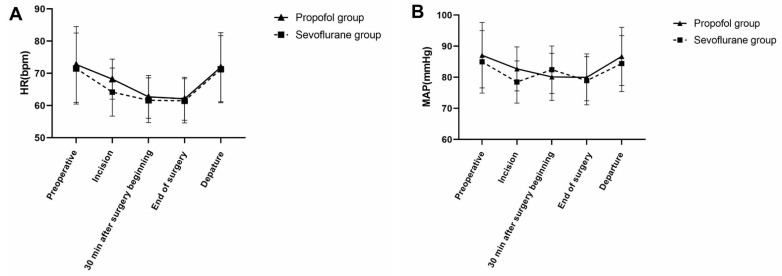
Heart rate values (bpm) and mean arterial pressure (MAP) values (mmHg) in both groups. MAP, mean arterial pressure; HR, heart rate. (**A**) No significant difference was noted in HR between the two groups, *p* > 0.05. (**B**) No significant difference was noted in MAP between the two groups, *p* > 0.05.

No differences were noted between the propofol and sevoflurane groups with regard to HR and the mean arterial pressure. All patients in the two groups exhibited stable hemodynamics during surgery (Figure 2).

### 3.4. Incidence, Severity, and Duration of POD

The overall incidence of POD among all participants in the current study was 21.60% (27 out of 125). The incidence of POD in the propofol and sevoflurane groups was 22.22% (14/63) and 20.97% (13/62), respectively (Table 3). No significant differences were noted between the propofol and sevoflurane groups with regard to the incidence, severity, and duration of POD (Table 3).

A total of 27 patients had at least one episode of delirium within the first five postoperative days. Among them, 74.07% (*n* = 20) had their first delirium diagnosis on postoperative day 1, whereas 85.19% (*n* = 23) of the participants had their first delirium diagnosis by the end of day 2, 92.59% (*n* = 25) by the end of day 3, 96.30% (*n* = 26) by the end of day 4, and 100% (*n* = 27) by the end of day 5, respectively. 

**Figure 3:**
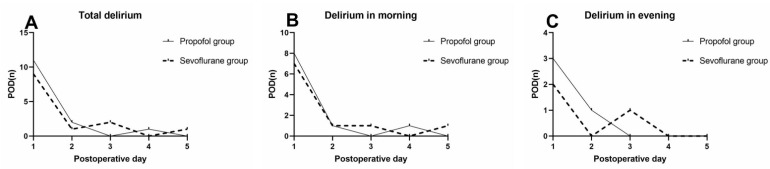
Incidence of first delirium event for patients during the first 5 postoperative days. (**A**) The incidence of the first episode of delirium. (**B**) Delirium in the morning; (**C**) delirium in the evening.

Furthermore, the exact time of the first delirium was compared among the different groups of patients. The data indicated that a higher number of patients experienced this condition in the morning than in the evening. Among them, 70.37% (*n* = 19) had their first delirium diagnosis in the morning and 29.63% (*n* = 8) in the evening. No significant differences were noted in the rate of the first diagnosis of delirium between the two groups (morning or afternoon of each postoperative day, Table 4 and Figure 3).

## 4. Discussion

No significant differences were noted in the incidence of POD between the participants of the propofol group (23.81%) and the sevoflurane groups (20.97%). Similar findings were noted for the duration and severity of POD between the propofol and sevoflurane groups. The present study suggested that anesthesia maintenance may have no definite effect on the POD of PD patients undergoing DBS surgery.

Although the administration of general anesthesia was initially considered to provide a safe, comfortable, and reversible state for surgery, the incidence of delirium in the early postoperative period and lasting cognitive dysfunction must be taken into consideration [14]. Therefore, several clinical or laboratory studies have been developed to explore the effect of general anesthesia on delirium.

Ishii et al. reported that propofol anesthesia was associated with a lower incidence of POD in elderly patients compared with that of patients under sevoflurane anesthesia [8]. This study reported that a faster emergence time and a lower number of adverse effects, such as nausea and vomiting, may both contribute to a reduction in the incidence of POD. It has also been shown that sevoflurane may induce neurotoxicity [15], which possibly influences the incidence of POD. However, these findings were revealed only under experimental conditions and it is unclear whether this neurotoxicity occurs in patients.

Zhang et al. also reported that propofol-based general anesthesia may decrease the incidence of delayed neurocognitive recovery in older adults following major cancer surgery compared with sevoflurane-based general anesthesia [12]. Three putative mechanisms were listed as follows. Firstly, volatile general anesthetics may produce neurotoxic effects, which may lead to cognitive impairment [21]. Secondly, the neuroinflammatory response may play an important role in the development of postoperative cognitive decline [22]. Volatile anesthetics tend to increase the expression of pro-inflammatory cytokines, which could aggravate the neuroinflammatory response provoked by surgical stress [22]. Thirdly, the pain intensity is less severe following propofol anesthesia compared with that noted following inhalational anesthesia [23], which may mitigate cognitive decline following surgery [24]. In the present study, no differences were noted in the pain scores between the two groups by effective pain control. 

However, contradictory studies have also been published. Whittington et al. reported that propofol could increase tau phosphorylation, which may also play an important role in the development of postoperative cognitive decline [25]. Mei et al. reported that propofol tended to cause a higher incidence of POD and a longer duration of this condition than sevoflurane [26]. The authors of this study speculated that the use of midazolam may be the possible reason contributing to the difference in the incidence and duration of POD between propofol- and sevoflurane-induced anesthesia [26]. Moreover, propofol may require a longer time to be eliminated from the body than sevoflurane [27,28]. Consequently, the authors of that study hypothesized that propofol may contribute, at least partially, to the higher incidence and longer duration of POD in the propofol group [26].

BIS monitoring is highly associated with sedation, consciousness, and memory, and aids in the determination of the extent of anesthesia. In addition, it is used to adjust the number of anesthetic agents administered and reduce the recovery time from general anesthesia [26]. Certain studies have shown that BIS monitoring during anesthesia aids the reduction of the sevoflurane requirement and results in more rapid emergence from general anesthesia [29]. Chan et al. reported that the titration of an anesthetic to maintain a BIS value between 40 and 60 during surgery could be used to avoid episodes of deep anesthesia, which was associated with a reduction in the risk of developing delirium [18]. Therefore, in the present study, the BIS value was maintained within 40 to 60.

POD is associated with a reduction in the intraoperative cerebral blood flow (CBF) [30]. Therefore, in the present study, the systemic blood pressure was kept higher than 90 mm Hg to ensure adequate CBF in both groups.

Compared with previous trials investigating the effects of propofol vs. inhalational anesthesia on POD, the present study had several distinct characteristics. Firstly, all the patients in the study received the same surgery by the same team. Secondly, all the patients in the present study received BIS monitoring to maintain a suitable depth of anesthesia. Thirdly, no differences were noted in the pain scores between the two groups. It was speculated that the aforementioned characteristics may explain the absence of significant differences noted in the incidence of POD between the participants in the propofol group and those in the sevoflurane group. Similar findings were noted for the duration days and severity of POD.

The present study contains several limitations. Firstly, it was a single-center study with a relatively small sample size, which demonstrated no significant differences in the incidence, severity, and duration of POD between the participants in the propofol- and sevoflurane-based anesthesia groups. Secondly, the participants only included patients who had DBS surgery. Thirdly, long-term outcomes including cognitive function, quality of life, and survival length were not investigated in the present study.

## 5. Conclusions

In conclusion, the present study indicated that the selection of the anesthesia maintenance regimen may not affect reduction in the risk of POD. The present study did not lead to an identifiable difference between the two anesthesia methods used for PD patients who received DBS surgery. Therefore, either choice of anesthesia (sevoflurane or propofol) can be selected. Surgical and patient-related factors may play more important roles in increasing the risk of POD. Future studies should investigate the contribution of modifiable surgical and patient-related factors in the reduction of the risk of POD.

## Figures and Tables

**Table 1:** Baseline characteristics of the patients in Propofol and Sevoflurane groups.

Variables (*n* = 125)	Propofol Group (*n* = 63)	Sevoflurane Group (*n* = 62)	*p*-Value
Age (y)	59.38 ± 9.27	59.45 ± 9.63	0.684
Male, *n* (%)	27 (41.27)	26 (41.94)	1.000
BMI, (kg/m^2^)	24.71 ± 4.00	23.22 ± 4.32	0.986
Education, (y)	11.08 ± 3.27	9.76 ± 3.43	0.648
ASA status, *n* (%)			
I	17 (26.98%)	28 (45.16%)	
II	41 (65.08%)	30 (48.39%)	0.103
III	5(7.94%)	4(6.45%)	
Preoperative comorbidities, *n* (%)			
Hypertension	5 (7.93%)	3 (4.83%)	0.732
Diabetes	5 (7.93%)	4 (6.45%)	1.000
Coronary artery disease	3 (4.76%)	2 (3.21%)	0.121
Stroke	1 (1.61%)	2 (3.22%)	0.619
Chronic smoking *	3 (4.76)	2 (3.23)	1.000
CDR score	0.12 (0.0.5)	0.12 (0.0.5)	0.795
IADL score	21.05 ± 1.67	21.05 ± 1.86	0.399
MoCA score	20.70 ± 3.69	20.32 ± 4.06	0.423
HAMA score	1.56 (0.19)	4.86 (0.19)	0.063
HAMD score	3.20 (0.22)	6.25 (0.19)	0.432

*: smoking of half a pack of cigarettes per day for at least 2 years. BMI, Body Mass Index; ASA, American Society of Anesthesiologists; CDR, Clinical Dementia Rating; IADL, Instrumental Activity of Daily Living; MoCA, Montreal Cognitive Assessment; HAMA, Hamilton anxiety; HAMD, Hamilton depression.

**Table 2:** The anesthesia and surgery characteristics of two groups.

Variables (*n* = 125)	Propofol Group (*n* = 63)	Sevoflurane Group (*n* = 62)	*p*-Value
Mean BIS value	45.97 ± 3.64	46.85 ± 3.78	0.184
Estimated blood loss, (mL)	72.06 ± 24.24	77.74 ± 23.15	0.183
Length of surgery, (min)	80.33 ± 32.20	76.11 ± 27.45	0.432
Length of anesthesia, (min)	124.00 ± 55.62	133.24 ± 51.69	0.338
Remifentanil, (mg)	1.29 ± 0.29	1.24 ± 0.25	0.284
Cisatracurium, (mg)	14.94 ± 2.18	14.34± 1.85	0.101
Open eyes time, (min)	5.71 ± 1.65	5.43 ± 1.51	0.314
Orientation time, (min)	10.21 ± 3.38	10.23 ± 2.93	0.973
Hypotension, *n* (%)	6 (9.52)	6 (9.68)	0.977
Bradycardia, *n* (%)	5 (7.93)	4 (6.45)	1.000
PONV, *n* (%)	7 (11.11)	9 (14.52)	0.569
VAS	3.78 ± 0.83	3.60 ± 0.664	0.182
Length of stay in hospital after surgery, (d)	10.35 ± 2.54	10.55 ± 2.57	0.664

PONV, postoperative nausea and vomiting; VAS, Visual Analog Scale.

**Table 3:** The characteristics of POD in two groups.

Variables (*n* = 125)	Propofol Group (*n* = 63)	Sevoflurane Group (*n* = 62)	*p*-Value
POD incidence, *n* (%)	14 (22.22)	13 (20.97)	0.865
MDAS	0 (0.18)	0 (0.18)	0.646
Duration of POD (days)	0 (0.3)	0 (0.3)	0.678

POD, postoperative delirium; MDAS, Memorial Delirium Assessment Scale.

**Table 4:** First delirium event for patients during the first 5 postoperative days.

Day Postoperative	Time	Propofol Group (*n* = 63)	Sevoflurane Group (*n* = 62)	Total (*n* = 125)	*p*-Value
1	Morning	8	7	15	0.863
	Evening	3	2	5	0.538
2	Morning	1	1	2	0.741
	Evening	1	0	1	0.519
3	Morning	0	1	1	0.481
	Evening	0	1	1	0.481
4	Morning	1	0	1	0.519
	Evening	0	0	0	/
5	Morning	0	1	1	0.481
	Evening	0	0	0	/

## Data Availability

All original datasets are available on request to the corresponding author.
